# Scaffold Free Bio-orthogonal Assembly of 3-Dimensional Cardiac Tissue via Cell Surface Engineering

**DOI:** 10.1038/srep39806

**Published:** 2016-12-23

**Authors:** Dmitry Rogozhnikov, Paul J. O’Brien, Sina Elahipanah, Muhammad N. Yousaf

**Affiliations:** 1 Department of Chemistry and Biology, York University, Toronto, M3J 1P3, Canada; 2 OrganoLinX Inc. Toronto, Canada

## Abstract

There has been tremendous interest in constructing *in vitro* cardiac tissue for a range of fundamental studies of cardiac development and disease and as a commercial system to evaluate therapeutic drug discovery prioritization and toxicity. Although there has been progress towards studying 2-dimensional cardiac function *in vitro*, there remain challenging obstacles to generate rapid and efficient scaffold-free 3-dimensional multiple cell type co-culture cardiac tissue models. Herein, we develop a programmed rapid self-assembly strategy to induce specific and stable cell-cell contacts among multiple cell types found in heart tissue to generate 3D tissues through cell-surface engineering based on liposome delivery and fusion to display bio-orthogonal functional groups from cell membranes. We generate, for the first time, a scaffold free and stable self assembled 3 cell line co-culture 3D cardiac tissue model by assembling cardiomyocytes, endothelial cells and cardiac fibroblast cells via a rapid inter-cell click ligation process. We compare and analyze the function of the 3D cardiac tissue chips with 2D co-culture monolayers by assessing cardiac specific markers, electromechanical cell coupling, beating rates and evaluating drug toxicity.

The generation of complex three-dimensional (3D) tissues with multiple cell types *in vitro* is the pinnacle of the lab on a chip, tissue engineering and artificial organ research fields^1^,^2^,^3^. Innovations in developing these types of tissues and assemblies are needed in order to revolutionize transplantation medicine, biomedical and drug discovery research^4^,^5^,^6^. Multidisciplinary approaches combining cell biology, bioengineering, polymer chemistry and regenerative medicine have resulted in the first wave of artificial tissue prototypes spanning pancreas, liver, kidney, skin and lung^7^,^8^,^9^,^10^. Although each organ has a specific architecture and comprise of multiple cell types, a special challenge in the artificial tissue field is the generation of cardiac tissue. The heart is a very cell dense muscular organ which pumps blood through arteries and veins of the circulatory system. Cardiovascular associated diseases are the leading cause of death globally and account for 40% of deaths in North America^11^. Furthermore, during the drug discovery process, cardiotoxicity is one of the major obstacles that result in the removal of drug candidates from clinical trials^12^. Therefore, production of 3-dimensional artificial cardiac tissues for fundamental studies of heart disease, transplantation and evaluation of drug toxicity is an important and intense area of research. A key design criteria to create a functional tissue *in vitro* is a method to assemble multiple cell types into a 3D structure^13^. The assembly method has to be efficient, inexpensive, non-immunogenic and non-cytotoxic. Techniques currently used for making 3D tissues include trapping cells in synthetic and natural polymer scaffolds. Natural scaffolds include collagen, matrigel, alginate, gelatin, chitosan as well as silk fibers and synthetic scaffolds include polymers such as polylactic acid, polyglycolic acid and their composites^14^,^15^,^16^. These materials have revolutionized tissue engineering research and allowed for 3D cell encapsulation and provide tunable mechanical properties such as controlled stiffness and elasticity. However, there are many parameters that need to be considered to make a scaffold-based tissue. These include: scaffold stability, porosity for oxygen and nutrients exchange, the rate of scaffold degradation, cytotoxicity of degradation by-products and potential inflammatory responses^17^. Furthermore, each scaffold type has a certain cross-linking density and therefore volume, and when mixed with cells, significantly reduces the cell density in the matrix/tissue hybrid material. This excluded volume from the scaffold creates a barrier for formation of high-density cell-cell junctions to establish intercellular communication. Such inter-connections are especially crucial for cardiac tissue, which requires a very high density of cells in order to enable long-range communication between cells via propagation of electrical signals to produce mechanical contractions that pump blood through long range synchronous beating. Cardiac tissue generation via polymer scaffolds *in vitro* is particularly challenging due to the much higher density of cells contained in the heart compared to any other organ (2–3% of heart tissue contains extracellular matrix while skin contains approximately 70%)^18^. In order to achieve synchronized long distance beating of tissue, the cells must have control of uninterrupted ion flow through their cytoplasms, which is only possible when they are physically interconnected through intercellular junction proteins called connexins^19^.

Herein, we present a scaffold-free method to generate high density 3- dimensional cardiac tissue consisting of multiple cardiac cell types. The self-assembly strategy combines for the first time, cell surface engineering and bio-orthogonal chemistry to rapidly click together 3 different cell types to generate a functional *in vitro* cardiac tissue. No external scaffold is used and the cells are the only building blocks of the generated cardiac tissue. We evaluate the self- assembled cardiac tissue with several assays including antibody markers, electromechanical beating rates, extracellular matrix production and influence of drugs on 2D and 3D synthesized cardiac tissues. To our knowledge, this is the first example of a 3-dimensional cardiac tissue that initially only consists of cells and does not contain any external supporting structure or scaffold.

## Results and Discussion

Cardiac tissue is one of the most cell dense organs due to the cardiomyocytes requirement to be physically connected in order to propagate electrical signals that result in large scale mechanical rhythmic beating with a synchronous pattern. Most of the heart organ is made up of cells with very little extracellular matrix proteins. For eg. other organs, such as aorta 25.7%, skin 64.5–72.1%, bone 15.1%, chordae (tendons) 77.1% contain much higher amounts of extracellular matrix and much less cell density than heart^18^.

**Figure 1:**
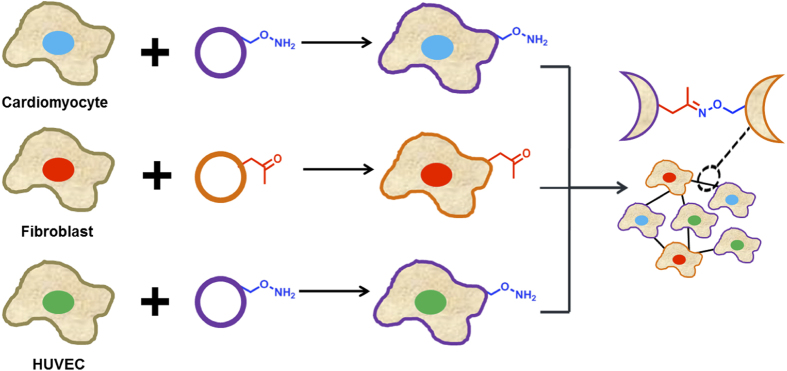
Schematic description for generating a scaffold free complex cardiac tissue by combining cell surface engineering and bio-orthogonal chemistry. The cells are initially treated with a rapid and mild liposome fusion method to install the bio-orthogonal groups onto the cell surface. Ketone and oxyamine groups on cell surface have been shown to rapidly click cells together via the oxime linkage and to form stable cell assemblies and tissues.

In order to generate scaffold free functional 3-dimensional cardiac tissue, we used the combination of liposome fusion, cell surface engineering and bio-orthogonal chemistry^20^,^21^,^22^,^23^. We have previously shown the rapid installation of bio-orthogonal ketone and oxyamine groups to a range of cell types via liposome fusion (ViaGlue)^24^,^25^,^26^. As ketone and oxyamine presenting cells come into contact the cells rapidly click together via the stable oxime ligation and assemble into spheroids and then tissues (Fig. 1). The interfacial oxime reaction is fast, chemoselective, occurs at physiological conditions (37 °C, pH 7) and requires no catalyst^27^,^28^,^29^. Furthermore, the resulting oxime bond has no side reactions with biomacromolecules, is bio-orthogonal and therefore does not interfere with native biological processes^30^.

The delivery of the bio-orthogonal groups to cells’ is based on rewiring the cell membranes with oxyamine and ketone moieties under mild conditions through the rapid process of liposome fusion. Ketone and oxyamine-functionalized lipids (*O*-dodecyloxyamine and dodecanone) together with widely used phospholipid palmitoyl-oleoyl phosphatidylcholine (POPC) and a cationic lipid 1,2 dioleoyl-3-trimethyammonium-propane (DOTAP) are incorporated into a liposome. When the bio-orthogonal liposomes (ViaGlue) are added to cells in cell culture, the liposomes rapidly fuse with the cellular membrane resulting in delivery of chemical functionality onto the cell surface. Membrane-engineered cardiac cells from different cell types are then clicked together to form a complex multicellular cardiac 3D tissue.

**Figure 2:**
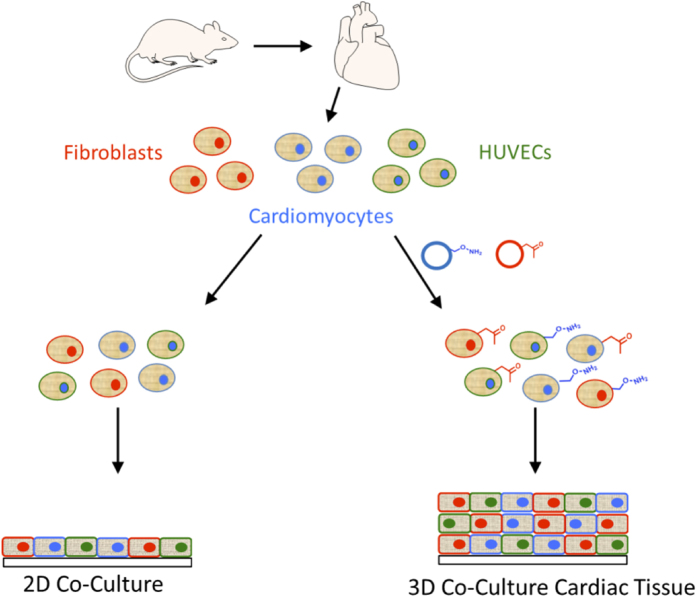
The schematic diagram representing the process of generating scaffold free 3 dimensional cardiac tissue via cell surface engineering and bio-orthogonal chemistry. The heart organ was first excised from new-born 24 h rat pups. The neonatal cardiomyocytes were isolated from the hearts. The fresh cardiomyocytes were immediately decorated with bio-orthogonal groups via liposome fusion and mixed with similarly engineered neonatal fibroblasts and human vascular endothelial cells (HUVECS) to form 3-dimensional cardiac tissue. The cells that were not treated with liposomes do not form a 3D tissue but instead form a standard 2D monolayer.

Cardiomyocytes, cardiac fibroblasts and human umbilical vein endothelial cells (HUVECs) were used to construct cardiac tissue^31^. In this work, primary cardiomyocytes were harvested from newborn Sprague Dawley rat pups (Fig. 2). Harvested cardiomyocytes together with HUVEC’s and fibroblasts were treated with ketone or oxyamine-containing liposomes. The liposome fusion process occurs in seconds to minutes and installs the bio-orthogonal groups onto the cell surface. It should be noted that the liposome fusion/bio-orthogonal delivery technology works on many mammalian cell types and is fast, mild and works within seconds to tailor cell surfaces on freshly harvested cardiomyocytes from rat hearts. The 3 surface-engineered cell types were mixed and rapidly clicked together and assembled into 3D tissues. The non-treated control cells (empty liposomes, or with unpaired bio-orthogonal groups) when mixed did not assemble and as expected formed only a single monolayer of cells in culture.

**Figure 3:**
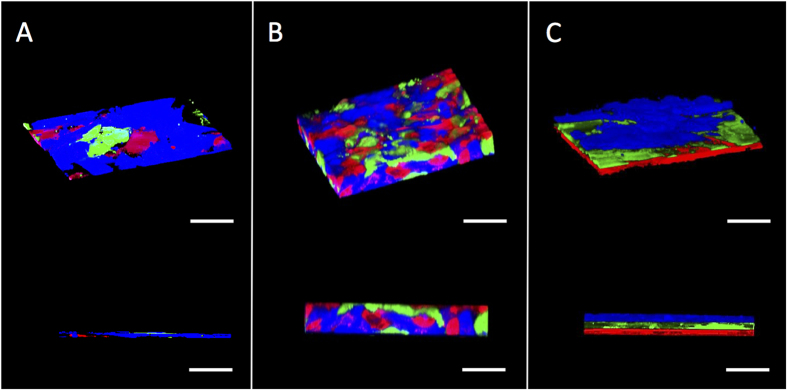
Confocal image representations of various 2D and 3D scaffold free cardiac tissue. Cardiomyocytes, HUVEC and fibroblast cells were live stained with fluorescent dyes (blue, green and red respectively). (**A**) The three cell types were mixed together and formed a single monolayer (10 μm thick). (**B**) The three cell types presented bio-orthogonal groups and when mixed clicked together and formed a random 3-dimensional multi-layer cardiac tissue (55 μm thick). (**C**) The three bio-orthogonal presenting cells were added sequentially to generate an oriented 3-dimensional cardiac tissue (20 μm thick). Scale bar = 60 μm.

Figure 3 shows confocal images of various 2 dimensional and 3 dimensional cardiac tissues. The ability to generate scaffold free thick 3D tissues as well as 3D tissues with controlled orientation was demonstrated via liposome fusion, cell surface engineering and bio-orthogonal chemistry. Prior to assembly, the cardiomyocytes HUVEC’s and fibroblasts were labeled with live-cell stain dyes, treated with liposomes and assembled into 3D tissues. Using oxime chemistry, it was possible to generate thick (55 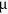
M) complex 3D tissues. In addition, this technology allows for assembly of tissues with defined multi-layer orientation when the different cell types are added in sequential order.

**Figure 4:**
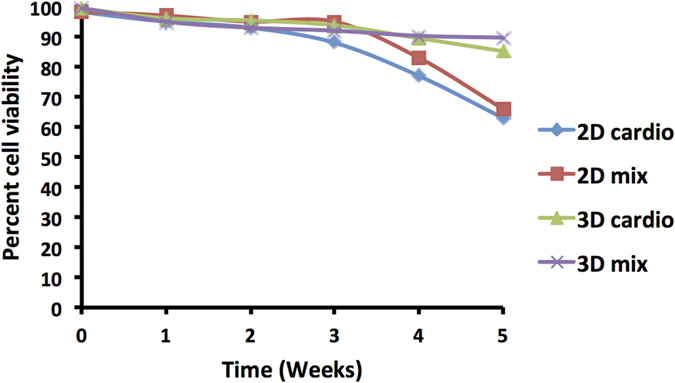
Cell viability in 2D and 3D tissues measured using propidium iodide viability assay. The various tissue constructs were stable and viable for several weeks. 2D Cardio = monolayer of cardiomyocytes. 2D mix = monolayer of mixed cardiomyocytes, fibroblasts and HUVEC cells. 3D cardio = 3 dimensional tissue comprising of only cardiomyocytes assembled via bio-orthogonal cell surface chemistry. 3D mix = 3 dimensional multilayer of mixed cardiomyocytes, fibroblasts and HUVEC cells assembled via bio-orthogonal cell surface chemistry.

Figure 3C shows three cell types may be oriented in layers allowing for strict pattern control. In the control sample, the cells were not treated with liposomes or treated with non-functionalized liposomes and as a result only 2D monolayers were obtained with an average thickness of 10 μM. The 3 dimensional tissues generated by the ViaGlue strategy were stable for several weeks (Fig. 4).

**Figure 5:**
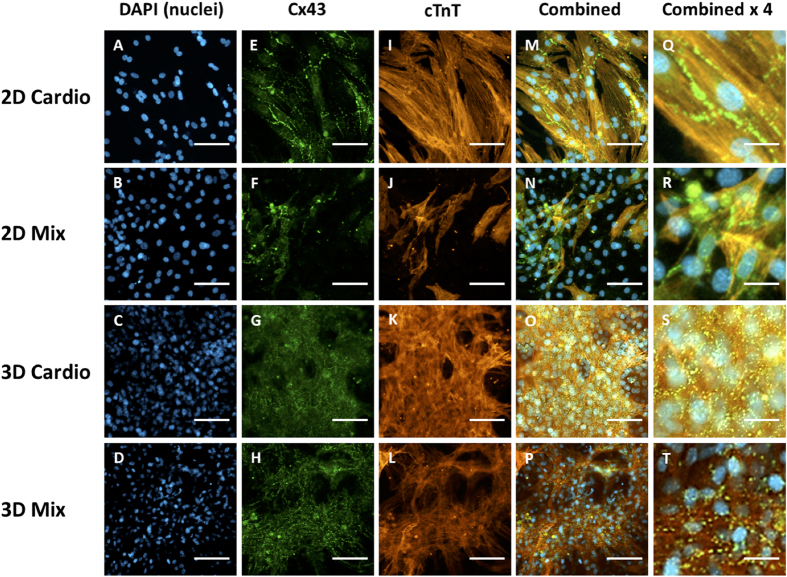
Fluorescent antibody staining for expression of cardiac-specific markers 96 h after tissue assembly. 2D Cardio = monolayer of cardiomyocytes. 2D mix = monolayer of mixed cardiomyocytes, fibroblasts and HUVEC cells. 3D cardio = 3 dimensional tissue comprising of only cardiomyocytes assembled via bio-orthogonal cell surface chemistry. 3D mix = 3 dimensional multilayer of mixed cardiomyocytes, fibroblasts and HUVEC cells assembled via bio-orthogonal cell surface chemistry. (**A–D**) DAPI nuclear staining. (**E–H**) Expression of cardiac gap-junction protein Connexin 43 (Cx 43) in 3D tissues and 2D control monolayers. (**I–L**) Expression of myocardial regulatory protein cardiac troponin T (cTnT). (**M–P**). Merged fluorescent images. (**Q–T**) Merged fluorescent images magnified four times. (**A–P**) Scale bar = 80 μM. (**Q–T**) Scale bar = 20 μM.

To evaluate the function of the scaffold free 3-dimensional assembled cardiac tissues, the various tissues were immunostained for the expression of cardiac-specific genetic markers (Fig. 5). Cardiac troponin T (cTnT) is responsible for contraction of cardiac muscle and is present in healthy tissue^32^. Connexin 43 (Cx 43) is a gap junction transmembrane protein that is expressed in working myocardium and facilitates propagation of calcium ions^33^. Both proteins are expressed in both 2D monolayers and 3D tissues. However, the 3D mixed tissues showed higher levels of expression in both cTnT and Cx 43, compared to the 2D mix monolayer. Both proteins are expressed in 3D cardiac and 3D mixed tissues demonstrating that the cardiomyocytes are well connected and the tissues are functional.

**Figure 6:**
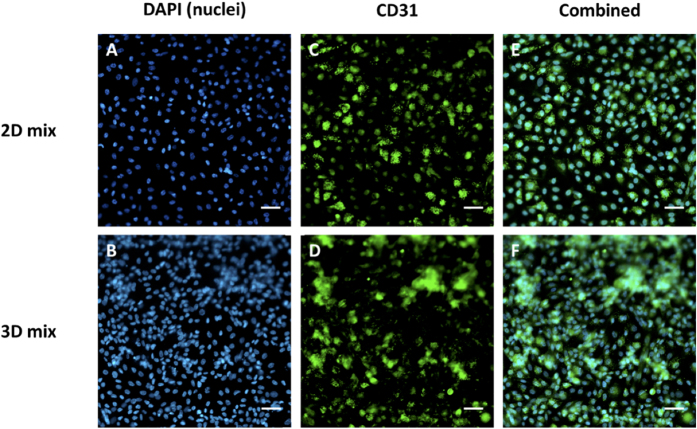
Fluorescent immunostaining for endothelial genetic marker CD31 expressed by HUVEC cells in 2D and 3D co-cultures 96 h after tissue assembly. (**A**,**B**) DAPI staining of cell nuclei. (**C**,**D**) Expression of CD 31 marker by HUVEC cells. (**E**,**F**) The merged fluorescent image. Scale bar = 60 μM.

To demonstrate the proper function of HUVEC cells in 3D tissue, the tissues were also stained for CD31, a genetic marker expressed on endothelial cells. CD31 is known to have various roles in vascular biology including angiogenesis, platelet function, and thrombosis. It is a mechano-sensor of endothelial cell response to fluid shear stress and it is involved in the regulation of leukocyte migration through venular walls^34^. Figure 6B shows high levels of expression for CD31 marker in 3D co-cultures compared to the 2D control. This demonstrates the proper functioning of HUVEC cells in 3D co-cultures.

**Figure 7:**
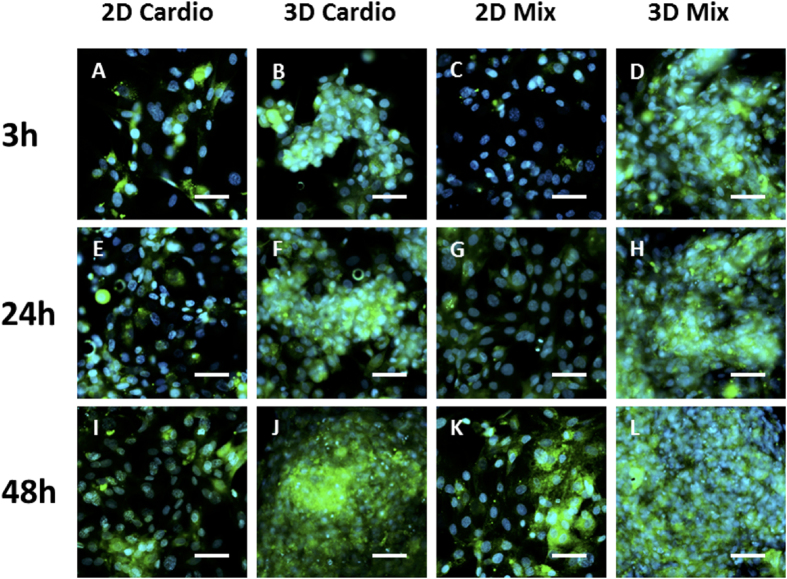
Fluorescent staining of collagen and elastin secreted over time by 2D monolayers and 3D tissues. The cells were assembled into tissues and stained for collagen and elastin with Col-F fluorescent dye at selected time points: 3 h (**A–D**), 24 h (**E–H**) and 48 h (**I–L**). Green is the fluorescent dye Col-F, blue is the DAPI nuclear stain. Scale bar = 80 μM.

Expression of extracellular matrix (ECM) by cells constituting myocardium is essential for proper functioning of cardiac tissue. The bio-orthogonal cell surface engineering strategy allows for the initial contact and assembly of the cells. However, over time, the cells in the assembly secrete their own extracellular matrix. The interfacial oxime bond click reaction initially nucleates the cell assembly process that does not naturally occur without scaffolds *in vitro*. To visualize production of ECM over time after the click cell assembly, the cells were stained with fluorescent probe markers specific for collagen and elastin (Fig. 7)^35^. Upon tissue assembly, the cells were treated with the dye marker and fixed with 4% formalin for various durations. The images show gradual secretion of ECM. It is observed that 3D tissues secrete more extracellular matrix than 2D tissues, even 3 h after cell assembly. After 48 h, the amount of ECM expressed is the highest, which demonstrates that the stability of the cell associations in the tissue is primarily through secretion of extracellular matrix and that the bio-orthogonal cell surface click chemistry is used primarily to initiate the assembly process. Over time, the cells excrete their own extracellular matrix, which then becomes the main adhesive glue that holds the cells and tissues together. Taken together, the bioorthogonal cell surface engineering method does not interfere with normal cell processes in tissue formation.

**Figure 8:**
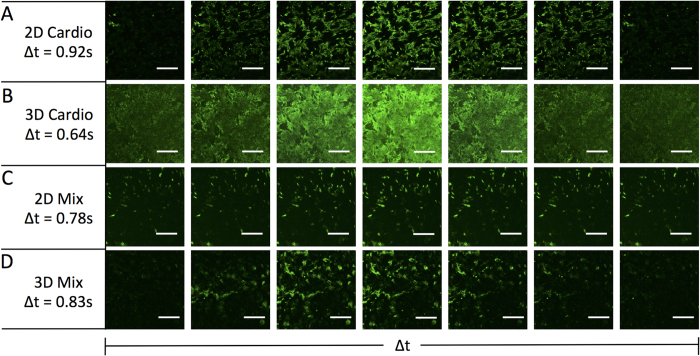
Native propagation of calcium across cardiac tissues without external stimulation. 48 h after assembly, the tissues were life-stained with Fluo-4 calcium binding fluorescent probe. The propagation of calcium wave was recorded as the video time series. The fluorescent pulse rate differed depending on the type of cell assembly and was 0.92 s for 2D cardiomyocytes (**row A**), 0.64 s for 3D cardiomyocytes (**row B**), 0.78 s for 2D mix. (**row C**) and 0.83 s for 3D mix (**row D**). Scale bar = 80 μM.

For cardiac tissue to function properly, the propagation of Ca^+2^, Na^+^ and K^+^ ions is essential for contractile activity throughout the myocardium^36^. The propagation of signal is also necessary to synchronize the electromechanical beating between the cells in the myocardium. Calcium staining was performed to measure the rate of signal wave through the various assembled 2D and 3D tissue constructs (Fig. 8). To demonstrate that there is an uninhibited calcium ion flow through the cell cytoplasms of interconnected cells via connexins, which are formed upon tissue assembly. After 48 h assembly, the various tissues were treated with fluo-4 – a calcium-binding fluorescent dye and visualized with fluorescence microscopy.

Snapshots were taken with frequency of 67 fps (frames per second) for 10 s, and combined into a video. The frequency of calcium pulses varied across the different tissue types: in 3D cardiomyocytes, the frequency was the greatest: every 0.64 s vs 0.83 s for 3D mixed tissues. The normal heart rate is approximately 0.80 s, which corresponds well with the mix 3D tissue generated. The intensity of fluorescence was the greatest in 3D cardiomyocytes monoculture and was due to the highest density of cardiomyocytes. In mixed 3D co-culture, the cardiomyocytes constitute only ~40% of all cells and the intensity of fluorescence is weaker. Overall, cardiac calcium signal propagation shows that there are proper cell-cell junctions between the cells in the tissue as well as a coordinated, simultaneous contraction of all cells. These scaffold free 3 dimensional tissues show intrinsic long range beating throughout the tissue without the application of an external voltage. This result is significant to show that the cells are tightly packed through the tissue and can conduct ion flow over large areas in order to beat synchronously as an entire unit. In our laboratory, we have found polymer or scaffold containing 3 dimensional cardiomyocyte tissues require an external applied current in order for all the cells to beat synchronously. We observed, in a hydrogel or collagen scaffold that there are local high density regions of beating cardiomyocytes that are independent populations and not synchronized to other regions due to the polymer matrix inhibiting physical contact between various populations of cells throughout the tissue (data not shown). Our method is scaffold free where the cells are self-assembled via cell surface engineering and therefore there is no need for outside stimulation for a synchronous beating of tissue.

Cardiac toxicity is a major cause for drug candidates to fail clinical trials. Standard *in vitro* cytotoxicity studies, utilize 2D monocultures of cardiomyocytes. 2D monolayers, however cannot recapitulate the complex 3D environment of myocardium and therefore, new *in vitro* 3D models are needed for accurate assessment of cardiac cytotoxicity of drug candidates. A major criteria for testing drugs in 3D cell culture versus 2D cell culture is that cells in three dimensions form multi-layers of cells, whereas cells grown in two dimensions form a single monolayer. When testing a drug in two dimensions, it needs only to diffuse a short distance across the cell membrane in order to reach its intended target cell. However, in three dimensions, the situation is more realistic to an organ and a drug needs to diffuse across multi-layers of cells. The diffusion across multi-layers of cells more closely mimics the challenges found in the human body or in cancer tissues in which a drug needs to diffuse through multiple layers of cells before it reaches its intended target. Furthermore, cells in three dimension tissues will form natural barriers to drugs, such as extracellular matrix and tight junctions that bind cells together and block or slow the diffusion of drugs, again making for a more realistic test model. For example, in a comparison of microarray data for gene expression of 3D cultured Hodgkin lymphoma line L1236 cells, of 2D cultured L1236 cells and of tumour samples from biopsy, gene expression patterns of the 3D cells were found to be more closely related to those of tumour samples than those cultured in two dimensions, with a marked difference for cell-substrate (2D) and cell-matrix (3D) adhesion molecules^37^.

**Figure 9:**
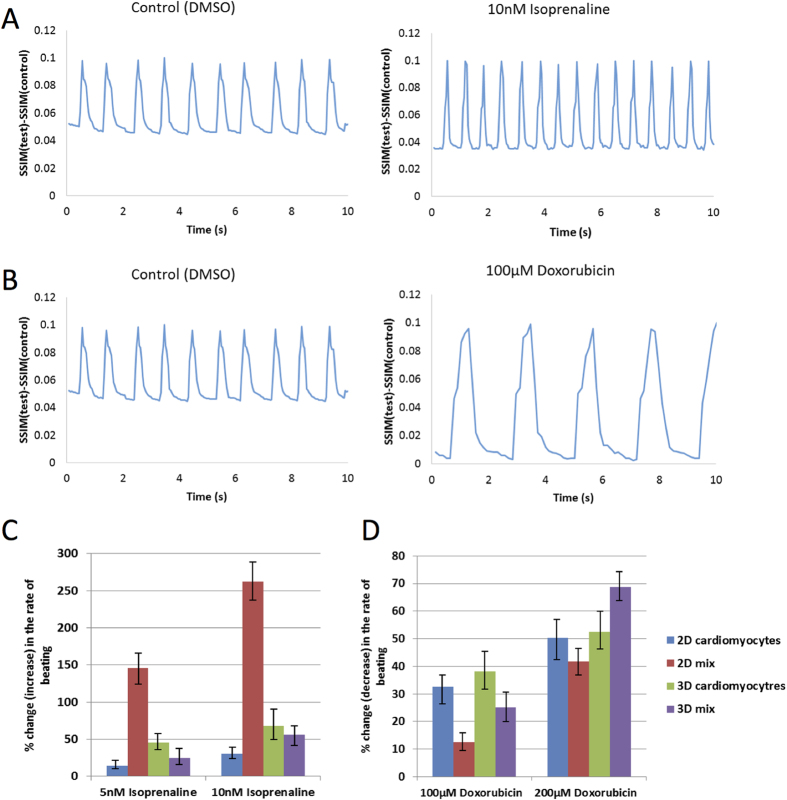
Chronotropic effects of cardiac tissues under treatment with varying concentrations of isoprenaline and doxorubicin. (**A**) A representative beating signal for 3D mix tissues. The increase in beating interval was in response to treatment with 10 nM isoprenaline. (**B**) A representative beating signal comparisons for 3D mix tissues. The beating interval decreased in response to treatment with 100 μM doxorubicin. (**C)** Percentage increase in beating rate for tissues treated with 5 nM and 10 nM isoprenaline. (**D)** Percentage decrease in beating rate for tissues treated with 100 μM and 200 μM doxorubicin. Error bars represent the standard error of the mean (s.e.m). n = 20, p < 0.05.

In this study, we evaluated the effect on beating rate of two chronotropic drugs, isoprenaline and doxorubicin on the various cardiac tissues (Fig. 9)^38^,^39^. Both drugs are known to affect the beating rate of cardiomyocytes. Isoprenaline is a drug used for treatment of bradycardia (slow heart rate) and asthma (acts as a bronchiadilator). Doxorubicin is an anticancer drug commonly used to treat stomach cancer, leukemia, as well as soft tissue sarcomas. The most profound side effect of doxorubicin and isoprenaline is cardiomyopathy, which leads to congestive heart failure. The tissues were treated with different concentrations of isoprenaline (5 nM and 10 nM) or doxorubicin (100 μM and 200 μM) for the duration of 25 min, followed by light microscopy videos to record the alteration in tissue beating. Figure 9A shows percent increase (for isoprenaline) or decrease (for doxorubicin) in beating rate of cardiomyocytes in 2D or 3D tissues compared to the control samples (treated with DMSO only). All tissues had an increase in beating rate in response to an increase of concentration of isoprenaline. As expected, the 2D cardiomyocytes were the most sensitive due to the accessibility of the drug to the cells in a monolayer compared to a 3D tissue. This phenomena of 2D sensitivity over 3D is well known and indicative that 2D monolayers are not realistic model systems of organ function. Doxorubicin treatment decreased the beating rate of cardiomyocytes and had the greatest effect on 3D tissue constructs. Cardiogram-like graphs were constructed to graphically represent the effect of isoprenaline and doxorubicin on cardiac beating rate (Fig. 9B). The image series were analyzed using ImageJ and a similarity score was assigned. The graph shows the increase in beating frequency for cells treated with isoprenaline and decrease in beating frequency for cells treated with doxorubicin. These results show the scaffold-free cardiac tissues beat spontaneously without external stimulation and react accordingly to known cardiomyocyte drug stimulants.

## Conclusion

In summary, we have used the combination of bio-orthogonal chemistry and cell surface engineering to program the rapid self-assembly of 3 different cell types into a functional 3-dimensional cardiac tissue. This click ligation method requires no polymers or extrinsic scaffold to trap or encapsulate cells. Cardiac tissue requires a high density of cells that are physically in contact in order to generate long range synchronous beating throughout the tissue. Significantly, the tissues generated by the ViaGlue liposome reagents were able to spontaneously beat synchronously throughout the entire tissue, due to high cell density and efficient cell contacts, without the requirement of external electrical stimulation. To evaluate the function of the various constructed cardiac tissues several cardiomyocyte antibody markers and drug toxicity assays were performed. Furthermore, we observe gradual production of extracellular matrix from the cells soon after tissue assembly via the inter cell click ligation. The ViaGlue strategy is general and capable of assembling a variety of cell types to generate a range of tissues. These tissues may be used for many applications including drug screening and as models for disease and infection as well as eventual cardiac patch *in vivo* applications. Many different cell types including stem cells may be used with the strategy and the inter cell click ligation is compatible with microfluidic and 3D printing technologies^23^,^24^,^25^,^26^,^27^,^28^,^29^,^30^. We believe the combination of liposome fusion, bio-orthogonal chemistry and cell surface engineering to tailor cell surfaces will have a significant impact on autocrine and paracrine signaling studies and for the development and evaluation of tissues for drug screening and therapeutic organ on a chip based biotechnology applications^40^,^41^,^42^,^43^,^44^.

## Materials and Methods

### Ethical Statement

Experimental animals were housed in a temperature controlled environment under 12 h light and 12 h dark conditions, and were fed ad-libitum. Animal facilities met Canadian Council on Animal Care guidelines and all protocols used were approved by the York University Animal Care Committee.

### Cardiomyocytes isolation

Neonatal cardiomyocytes were isolated from newborn 1–2 day old Sprague Dawley rat pups. The pups were euthanized via spinal dislocation and their hearts were excised. The atrial parts of the hearts were removed and the hearts were cut in half to remove blood fluid. The procedure was performed in CBFHH buffer: 137 mM NaCl, 20 mM Hepes, 0.44 mM KH_2_PO_4_, 5.6 mM dextrose, 5.4 mM KCl, 0.8 mM MgSO_4_, pH 7.4. The hearts were then trimmed with surgical scissors into small pieces (1–1.5 mm^2^). The cardiac tissue was digested via serial digestion in enzymatic buffer: CBFHH buffer + 1.5 mg/ml of trypsin. Digestion was performed in a series of steps, 5 min each at 37 °C. The digests were collected into a test tube containing 5 mL of concentrated FBS. The cells were centrifuged at 800 rpm for 5 min. The cells were placed in a flask containing Ham’s F-12 Medium (10% FBS, 1% penicillin/streptomycin) medium and incubated for 45 min, to allow cardiac fibroblasts present in the tissue to adhere to the bottom of the flask. The medium containing purified cardiomyocytes was then transferred to a separate flask.

### Tissue culture

Human umbilical vein endothelial cells (HUVEC) were purchased from ATCC (Canada). The cells were cultured on round 10 cm plastic tissue culture plates. The medium used was F-12K Medium (Kaighn’s Modification of Ham’s F-12 Medium) with 10% fetal bovine serum (FBS), 0.1 mg/ml heparin (Sigma), 0.05 mg/ml endothelial cell growth supplement (ECGS) (Sigma) and 1% penicillin/streptomycin. The cells were incubated at 37 °C, 5% CO_2_, the medium was replaced every 48 h and the cells were passaged upon reaching 90% confluence. Human neonatal dermal fibroblasts were purchased from ATCC (Canada). The fibroblasts were cultured in Dulbecco’s modified Eagle high glucose medium (DMEM) with 10% FBS and 1% penicillin/streptomycin.

### Preparation of liposomes

To prepare oxyamine and ketone-tethered liposomes, chloroform solutions of palmitoyl-oleoyl phosphatidylcholine (POPC), 1,2 dioleoyl-3-trimethyammonium-propane (DOTAP) were mixed with *O*-dodecyloxyamine (for oxyamine-tethered liposomes) or dodecanone (for ketone-tethered liposomes on the following ratios: POPC (430 μL,10 mg/mL in CHCl_3_ at 86 mol%); DOTAP (10 μL, 10 mg/mL in CHCl_3_ at 2 mol%); and *O*-dodecyloxyamine or dodecanone (60 μL, 10 mM in CHCl_3_ at 12 mol%). The mixtures of lipids were thoroughly dried and then re-suspended in 3 ml of phosphate-buffered saline (PBS). The suspension was then sonicated with a tip sonicator at the power of 20 Watts for 20 min until it was clear.

### Tissue assembly

Isolated cardiomyocytes were centrifuged at 600 rpm for 5 min and the medium was discarded. HUVECs and human neonatal fibroblasts were allowed to achieve 85–90% confluence prior to tissue assembly and were trypsinized and centrifuged at 800 rpm for 5 min. 500 μl of oxyamine- or ketone-tethered liposomes were added to the cell pellet, the cells were re-suspended and incubated at 37 °C for 5 min. The cells were then washed with PBS and re-centrifuged. The PBS was discarded and the oxyamine- and ketone- labeled cardiomyocytes, HUVECs and fibroblasts were mixed in a small volume of combined (1:1) Ham’s F12: F-12K Kaighn’s medium with heparin and ECGF. The total cell concentration was 5 × 10^6 ^cells/ml. 50 μL drops of the concentrated re-suspended cell solutions were then placed on 1 cm^2^ nitrocellulose-coated glass slides and given a slight shake to induce cell assembly. Each mixed (co-culture) 2D or 3D sample contained 1 × 10^5^ cardiomyocytes, 1 × 10^5^ HUVECs and 5 × 10^4^ fibroblasts. Each 2D and 3D cardiomyocyte-only culture (monoculture) contained 1 × 10^5^ cells. The cells were then incubated at 37 °C and 5% CO_2_ for 24 h to achieve full spreading into tissues. Upon spreading of cells, fresh medium was added to the plates. Cells in the control samples were treated with non-functionalized liposomes.

### Immunohistochemistry and confocal microscopy

Prior to tissue assembly, cells were incubated in serum-free medium containing fluorescent live stain (Life Technologies) dyes. Neonatal rat cardiomyocytes, HUVECS, and human neonatal fibroblasts were treated with 25 μM CellTracker™ Blue CMAC (7-amino-4-chloromethylcoumarin), 25 μM CellTracker™ Green CMFDA (5-chloromethylfluorescein diacetate), and 25 μM CellTracker^TM^ Red CMTPX respectively. The cells were incubated at 37 °C for 45 min, and then washed thoroughly with PBS and incubated in serum-free medium for an additional 45 min. The cells were then assembled into 3D tissues and incubated for 24 h. Subsequently, the tissues were fixed with 4% formalin for 10 min and visualized with LSM-700 (Zeiss) confocal microscope.

### Immunostaining

The cells were fixed with 4% formalin for 10 min at room temperature and washed 4 times with PBS. To permeabilize the cell membrane, the samples were incubated in cold (−20 °C) 90% methanol for 5 min at 4 °C. Methanol was decanted and the cells were rinsed 3 times with PBS. The samples were treated with a blocking solution of 5% FBS in PBS at 37 °C for 60 min. While blocking, the dilution of primary monoclonal antibodies were prepared in 5% FBS. The 500–600X dilutions of anti-connexin 43, anti-cardiac troponin T or anti-CD31 primary antibodies (Abcam) were used. The blocking solution was removed and the samples were incubated with the solution of primary antibodies at 4 °C for 12 h. The solution was aspirated and the samples were washed 3 times with PBS. Following that, the samples were incubated in solution 900X-diluted FITC- and TRITC-conjugated secondary antibodies in the dark for 2 h at room temperature. The samples were washed 3 times with PBS and visualized under fluorescent microscope.

### Fluorescent staining for Collagen and Elastin

To observe the secretion of ECM over time for the various assembled 2D and 3D tissues, the samples were treated with Col-F fluorescent probe (Immunochemistry Technologies, MN), which has an affinity for collagen and elastin. The stock solution of Col-F (20 mM) was prepared in DMSO. The medium was replaced with the medium containing 20 μM Col-F. The cells were incubated at 37 °C for 60 min, after which they were washed thoroughly with PBS and fixed with 4% formalin for 10 min with subsequent staining with DAPI for visualization of cellular nuclei. The samples were visualized under fluorescent microscope with excitation wavelength of 488 nm and emission of 520 nm.

### Fluorescent Calcium Imaging

The tissues were incubated with 5 μM of the calcium-sensitive dye Fluo-4 AM (Life Technologies) for 20 min at 37 °C. The samples were washed with Tyrode salt solution for 20 min and calcium transients were recorded using fluorescent imaging with excitation wavelength of 488 nm. Recording was performed for the duration of 10 s with the frequency of 67 frames per second (fps).

### Cardiotoxicity testing

72 h upon tissue assembly, the tissue response to cytotoxic effects of two drugs, isoprenaline and doxorubicin (Sigma) were evaluated. The stock solutions of the drugs were prepared in DMSO. Each drug was dissolved in the culture medium and added to the slide containing assembled tissue. The control samples were treated with DMSO containing medium. The samples were incubated for 25 min with the corresponding drug and the change in the beating rate relative to the control was measured. Twenty independent experiments were performed for each drug to obtain statistically reliable data.

### Real time image processing

To measure the variances in cardiomyocyte beating in response to drug treatment, a movie of beating cardiomyocytes in 3D co-cultures was captured under a light microscope. The series of images that make up the movie were analyzed using ImageJ software plugin SSIM index. This program compares two images and assigns a similarity score (SSIM index). Identical images receive a SSIM index of 1, and completely different – a similarity index of 0. The image with the tissue being completely contracted was taken for reference and assigned a similarity index of 1 and all other images of tissue undergoing different stages of contraction was measured against the reference image. As a result, when the tissue fully contracted, its SSIM index approached a value of 1.0, and as it was relaxing, the SSIM index was decreasing. To eliminate the noise signal, the image series of non-moving fixed tissues were recorded. The average SSIM measurement of non-moving fixed tissues was taken as the control and was subtracted from each result. The resulting number was plotted and resulted in a cardiogram-like graph.

## Additional Information

**How to cite this article:** Rogozhnikov, D. *et al*. Scaffold Free Bio-orthogonal Assembly of 3-Dimensional Cardiac Tissue via Cell Surface Engineering. *Sci. Rep.* **6**, 39806; doi: 10.1038/srep39806 (2016).

**Publisher's note:** Springer Nature remains neutral with regard to jurisdictional claims in published maps and institutional affiliations.

## References

[b1] Bajaj P., Schweller R. M., Khademhosseini A., West J. L. & Bashir R. 3D Biofabrication Strategies for Tissue Engineering and Regenerative Medicine. Annu. Rev. Biomed. Eng. 16, 247–276 (2014). 24905875 10.1146/annurev-bioeng-071813-105155 PMC4131759

[b2] Vunjak-Novakovic G. . Challenges in cardiac tissue engineering. Tissue Eng. Part B. Rev. 16, 169–187 (2010). 19698068 10.1089/ten.teb.2009.0352 PMC2946883

[b3] Lutolf M. P. & Hubbell J. A. Synthetic biomaterials as instructive extracellular microenvironments for morphogenesis in tissue engineering. Nat. Biotechnol. 23, 47–55 (2005). 15637621 10.1038/nbt1055

[b4] Uygun B. E. . Organ reengineering through development of a transplantable recellularized liver graft using decellularized liver matrix. Nat. Med. 16, 814–820 (2010). 20543851 10.1038/nm.2170 PMC2930603

[b5] Layered long-term co-culture of hepatocytes and endothelial cells on a transwell membrane: toward engineering the liver sinusoid. Biofabrication 5, 045008 (2013). 24280542 10.1088/1758-5082/5/4/045008 PMC3935322

[b6] Page H., Flood P. & Reynaud E. G. Three-dimensional tissue cultures: current trends and beyond. Cell Tissue Res. 352, 123–131 (2013). 22729488 10.1007/s00441-012-1441-5

[b7] Rustad K. C., Sorkin M., Levi B., Longaker M. T. & Gurtner G. C. Strategies for organ level tissue engineering. Organogenesis. 6, 151–157 (2010). 21197216 10.4161/org.6.3.12139 PMC2946046

[b8] Khetani S. R. & Bhatia S. N. Microscale culture of human liver cells for drug development. Nat. Biotechnol. 26, 120–126 (2008). 18026090 10.1038/nbt1361

[b9] Petersen T. H. . Tissue-engineered lungs for *in vivo* implantation. Science. 329, 538–541 (2010). 20576850 10.1126/science.1189345 PMC3640463

[b10] Groeber F., Holeiter M., Hampel M., Hinderer S. & Schenke-Layland K. Skin tissue engineering — *In vivo* and *in vitro* applications. Adv. Drug Deliv. Rev. 63, 352–366 (2011). 21241756 10.1016/j.addr.2011.01.005

[b11] Roger V. L. . Heart disease and stroke statistics–2011 update: a report from the American Heart Association. Circulation 123, e18–e209 (2011). 21160056 10.1161/CIR.0b013e3182009701 PMC4418670

[b12] Pereira G. C. . Drug-induced Cardiac Mitochondrial Toxicity and Protection: From Doxorubicin to Carvedilol. Curr. Pharm. Des. 17, 2113–2129 (2011). 21718248 10.2174/138161211796904812

[b13] Emmert M. Y., Hitchcock R. W. & Hoerstrup S. P. Cell therapy, 3D culture systems and tissue engineering for cardiac regeneration. Adv. Drug Deliv. Rev. 69–70, 254–269 (2014). 10.1016/j.addr.2013.12.004 24378579

[b14] Shachar M., Tsur-Gang O., Dvir T., Leor J. & Cohen S. The effect of immobilized RGD peptide in alginate scaffolds on cardiac tissue engineering. Acta Biomater. 7, 152–162 (2011). 20688198 10.1016/j.actbio.2010.07.034

[b15] Chen J. L. . Efficacy of hESC-MSCs in knitted silk-collagen scaffold for tendon tissue engineering and their roles. Biomaterials. 31–36, 9438–9451 (2010). 10.1016/j.biomaterials.2010.08.011 20870282

[b16] Eng D., Caplan M., Preul M. & Panitch A. Hyaluronan scaffolds: a balance between backbone functionalization and bioactivity. Acta Biomater. 6, 2407–2414 (2010). 20051273 10.1016/j.actbio.2009.12.049

[b17] Zhang H., Zhou L. & Zhang W. Control of scaffold degradation in tissue engineering: a review. Tissue Eng. Part B. Rev. 20, 492–502 (2014). 24547761 10.1089/ten.TEB.2013.0452

[b18] Neuman R. E. & Logan M. A. The determination of collagen and elastin in tissues. J Biol Chem. 2, 549–556 (1950). 14794650

[b19] You J.-O., Rafat M., Ye G. J. C. & Auguste D. T. Nanoengineering the heart: conductive scaffolds enhance connexin 43 expression. Nano Lett. 11, 3643–3648 (2011). 21800912 10.1021/nl201514a

[b20] Bertozzi C. R. A decade of bioorthogonal chemistry. Acc. Chem. Res. 44, 651–653 (2011). 21928847 10.1021/ar200193f PMC4408923

[b21] McKay C. S. & Finn M. G. Click chemistry in complex mixtures: bioorthogonal bioconjugation. Chem. Biol. 21, 1075–1101 (2014). 25237856 10.1016/j.chembiol.2014.09.002 PMC4331201

[b22] Patterson D. M., Nazarova L. A. & Prescher J. A. Finding the right (bioorthogonal) chemistry. ACS Chem. Biol. 9, 592–605 (2014). 24437719 10.1021/cb400828a

[b23] O’Brien P. J., Luo W., Rogozhnikov D., Chen J. & Yousaf M. N. Spheroid and Tissue Assembly via Click Chemistry in Microfluidic Flow. Bioconjug. Chem. 26, 1939–1949 (2015). 26267284 10.1021/acs.bioconjchem.5b00376

[b24] Dutta D., Pulsipher A., Luo W. & Yousaf M. N. Synthetic chemoselective rewiring of cell surfaces: generation of three-dimensional tissue structures. J. Am. Chem. Soc. 133, 8704–8713 (2011). 21561150 10.1021/ja2022569

[b25] Luo W., Pulsipher A., Dutta D., Lamb B. M. & Yousaf M. N. Remote control of tissue interactions via engineered photo-switchable cell surfaces. Sci. Rep. 4, 6313 (2014). 25204325 10.1038/srep06313 PMC4159631

[b26] Park S., Westcott N. P., Luo W., Dutta D. & Yousaf M. N. General chemoselective and redox-responsive ligation and release strategy. Bioconjug. Chem. 25, 543–551 (2014). 24559434 10.1021/bc400565y PMC3983135

[b27] Dutta D., Pulsipher A., Luo W., Mak H. & Yousaf M. N. Engineering cell surfaces via liposome fusion. Bioconjug. Chem. 22, 2423–2433 (2011). 22054009 10.1021/bc200236m

[b28] Elahipanah S., Radmanesh P., Luo W., O’Brien P. J., Rogozhnikov D. & Yousaf M. N. Rewiring Gram-Negative Bacteria Cell Surfaces with Bio-Orthogonal Chemistry via Liposome Fusion. Bioconjugate Chem. ASAP, doi: 10.1021/acs.bioconjchem.6b00073 (2016). 27019118

[b29] Luo W. . A Dual Receptor and Reporter for Multi-Modal Cell Surface Engineering. ACS Chem. Biol. 10, 2219–2226 (2015). 26204094 10.1021/acschembio.5b00137

[b30] Pulsipher A., Dutta D., Luo W. & Yousaf M. N. Cell surface engineering by a conjugation and release approach based on the formation and cleavage of oxime linkages upon mild electrochemical oxidation and reduction. Angew. Chem. Int. Ed. 53, 9487–9492 (2014). 10.1002/anie.201404099 25045145

[b31] Vuorenpää H. . Novel *in vitro* cardiovascular constructs composed of vascular-like networks and cardiomyocytes. In Vitro Cell. Dev. Biol. Anim. 50, 275–286 (2014). 24163159 10.1007/s11626-013-9703-4

[b32] Sehnert A. J., Huq A., Weinstein B. M., Walker C., Fishman M. & Stainier D. Y. Cardiac troponin T is essential in sarcomere assembly and cardiac contractility. Nat Genet. 1, 106–110 (2002). 10.1038/ng875 11967535

[b33] Michela P., Velia V., Aldo P. & Ada P. Role of connexin 43 in cardiovascular diseases. Eur J Pharmacol. 768, 71–76 (2015). 26499977 10.1016/j.ejphar.2015.10.030

[b34] Pusztaszeri M. P., Seelentag W. & Bosman F. T. Immunohistochemical expression of endothelial markers CD31, CD34, von Willebrand factor, and Fli-1 in normal human tissues. J Histochem Cytochem. 4, 385–395 (2006). 10.1369/jhc.4A6514.2005 16234507

[b35] Biela E. . Col-F, a fluorescent probe for *ex vivo* confocal imaging of collagen and elastin in animal tissues. Cytometry A. 6, 533–539 (2013). 10.1002/cyto.a.22264 PMC3671577 23404939

[b36] Aiba, T. & Tomaselli G. F. Electrical remodeling in the failing heart. Curr Opin Cardiol. 25, 29–36 (2010). 19907317 10.1097/HCO.0b013e328333d3d6 PMC2855498

[b37] Birgersdotter A. . Three-dimensional culturing of the Hodgkin lymphoma cell-line L1236 induces a HL tissue-like gene expression pattern. Leukemia & Lymphoma 48, 2042–2053 (2007). 17917972 10.1080/10428190701573190

[b38] Wallace K. B. Doxorubicin-induced cardiac mitochondrionopathy. Pharmacol Toxicol. 93, 105–115 (2003). 12969434 10.1034/j.1600-0773.2003.930301.x

[b39] Fink C., Ergün S., Kralisch D., Remmers U., Weil J. & Eschenhagen T. Chronic stretch of engineered heart tissue induces hypertrophy and functional improvement. FASEB J. 14, 669–679 (2000). 10744624 10.1096/fasebj.14.5.669

[b40] Stevens M. M. & George J. H. Exploring and engineering the cell surface interface. Science 310, 1135–1138 (2005). 16293749 10.1126/science.1106587

[b41] Gartner Z. J. & Bertozzi C. R. Programmed assembly of 3-dimensional microtissues with defined cellular connectivity. Proc. Natl. Acad. Sci. USA 106, 4606–4610 (2009). 19273855 10.1073/pnas.0900717106 PMC2660766

[b42] Gou M. . Bio-inspired detoxification using 3D-printed hydrogel nanocomposites. Nat. Commun. 5, 3774 (2014). 24805923 10.1038/ncomms4774 PMC4024742

[b43] Kelm J. M. . A novel concept for scaffold-free vessel tissue engineering: self-assembly of microtissue building blocks. J. Biotechnol. 148, 46–55 (2010). 20223267 10.1016/j.jbiotec.2010.03.002

[b44] Zorlutuna P. . Microfabricated biomaterials for engineering 3D tissues. Adv. Mater. 24, 1782–1804 (2012). 22410857 10.1002/adma.201104631 PMC3432416

